# An Unusual Case of Hypoproteinemia in Childhood: Keep in Mind Trichobezoar

**DOI:** 10.3389/fped.2020.00082

**Published:** 2020-03-04

**Authors:** Mariangela Stinco, Alessandra Montemaggi, Bruno Noccioli, Massimo Resti, Salvatore Grosso, Sandra Trapani

**Affiliations:** ^1^Pediatric Section, Department of Health Sciences, Meyer Children's University Hospital, University of Florence, Florence, Italy; ^2^Pediatric Surgery Unit, Meyer Children's University Hospital, University of Florence, Florence, Italy; ^3^Pediatric Neurology-Immunology and Endocrinology Unit, Le Scotte Hospital, University of Siena, Siena, Italy

**Keywords:** Rapunzel Syndrome, trichobezoar, hypoproteinemia, protein-losing enteropathy, oedema

## Abstract

Protein-losing enteropathy (PLE) is a rare condition characterized by protein loss through the gastrointestinal tract, leading to hypo-proteinemia. Patients may be asymptomatic or present with variety of complications of hypoproteinemia (e.g., oedema, ascites, pleural, and cardial effusions). We describe a case report of a young girl suffering from behavioral disorder since childhood who presented with generalized oedema, hypoproteinaemia, and microcytic hypochromic anemia. In addition, the girl had an intervention for jejunal atresia and intestinal malrotation in her past medical history. Upper gastrointestinal endoscopy revealed a trichobezoar extending from stomach into the small bowel, thus classified as Rapunzel Syndrome (RS), causing mechanical obstruction of intestinal lumen and intestinal lymphatic drainage resulting in a protein-losing enteropathy (PLE). Trichobezoar was successfully removed by a surgical laparotomy resulting in resolution of symptoms and normalization of biochemical parameters. Possibly, previous surgery might have had an influence on intestinal dysmotility and trichobezoar formation. PLE is a very rare presenting symptom of RS, developing as result of intestinal obstruction caused by large trichobezoars. RS has to be considered in patients, especially adolescents, suffering from behavior disorder as trichotillomania and trichophagia. Surgical removal and nutritional supplementation are the gold treatment of large trichobezoar.

## Introduction

Protein-losing enteropathy (PLE) is a rare intestinal disorder characterized by protein loss through the gastrointestinal tract, resulting in hypoproteinemia. The disease can be caused by abnormalities of the intestinal lymphatic system or impaired integrity of the mucosa; the diagnosis is suspected from clinical manifestations, but it can be confirmed by increased fecal level of alfa-1-antitrypsin. PLE and oedema may be very rarely onset manifestation of trichobezoar extending from stomach into the small bowel, therefore classified as Rapunzel Syndrome (RS) (1, 2). In fact, as the trichobezoar enlarges can cause intestinal plasma protein loss through both mechanical obstruction of intestinal lymphatic drainage and mucosal inflammatory injury (3). We report a case of small bowel obstruction and protein-losing enteropathy in a young girl with Rapunzel Syndrome. For data collection and publication, written informed consent was obtained from the parents of the patient.

## Case Report

A 7-year-old Caucasian girl presented to the Emergency Department of a local hospital with a 2 weeks history of lower limb oedema, then widespread in the pubic region, abdomen, and eyelid associated with diarrhea and cough during the previous days. Her past medical history included jejunal atresia type I, neonatal giant cell hepatitis, intestinal malrotation, gallbladder's agenesis, ventricular septal defect, previous episodes of angioedema and urticaria, short stature, congenital nystagmus. The mother also reported that her child suffered from trichotillomania and trichophagia since she was 3 years old.

**Figure 1:**
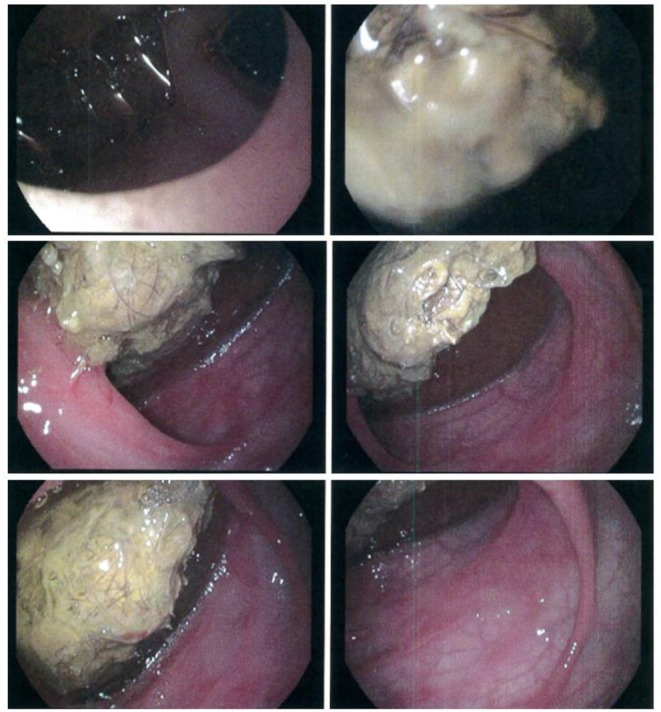
Gastrointestinal endoscopy: a large tricobezoar occupying lumen of distal duodenum.

On admission, the patient was malnourished (body weight 18.7 kg and height 112 cm, both at the 3rd percentile for age), while on physical examination, her abdomen was tender with generalized oedema and the liver was palpable at the costal arch. Blood exams showed microcytic hypochromic anemia [hemoglobin (Hb) 7.5 g/dl, MCV 68,7 fl, MCH 19 pg], hypoproteinaemia (total protein 3.3 g/dl) and severe hypoalbuminemia (albumin 1.8 g/dl); creatinine, transaminases, and urinalysis were normal. She underwent red blood cells transfusion and repeated intravenous (IV) albumin administrations (0.5 g/kg body weight) with improvement of laboratory tests: Hb 10.3 g/dl and albumin 2.9 g/dl. Abdominal ultrasound demonstrated ascites, hypoperistaltic small-bowel loops, and distension of ascending colon. These findings were further investigated by oral contrast study that showed dilated small-bowel loops and distension of the distal ileum where a slow transit was noted, and by upper endoscopy revealing a trichobezoar with distal duodenum and jejunum stenosis (Figure 1). Although generally ineffective, endoscopic extraction of trichobezoar was tried, unsuccessfully. Therefore, she was referred to our hospital for surgical evaluation.

Initially, she was transferred to our general pediatrics ward for assessment and nutrition support. Biochemical testing showed low immunoglobulin G level (378 mg/dl, with normal value for age 540-1330 mg/dl), persistent hypoalbuminemia (serum albumin 2.9 g/dl with total protein 5.6 g/dl) which was managed with IV albumin (at the dose of 1 g/kg body weight) and reduced prothrombin time (PT) 69% and partial thromboplastin time (PTT) 29 s successfully treated with vitamin K administration (5 mg). The 24-h urine sample analysis, performed to exclude renal protein loss, was normal. Stool levels of alpha-1-antitrypsin, a sensitive marker for diagnosing loss of plasma proteins from the gastrointestinal tract, were significantly increased 813 mcg/g (normal value <248 mcg/g).

**Figure 2:**
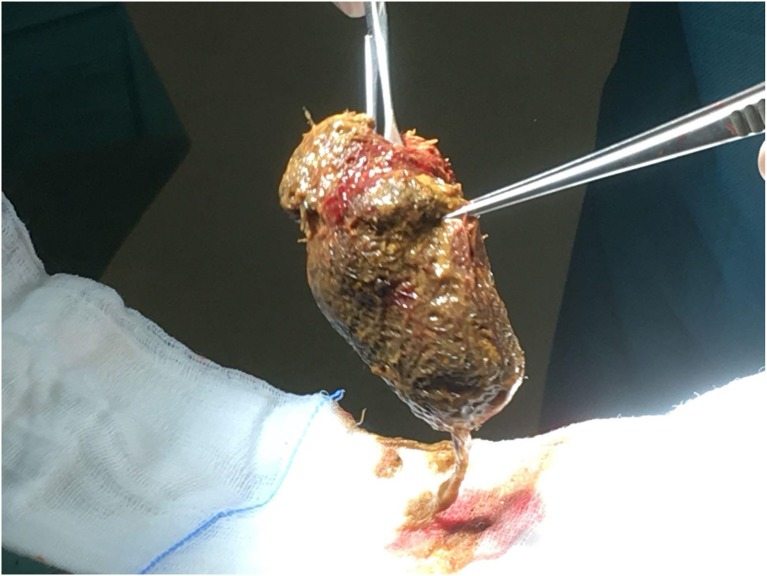
Large intra-luminal solid mass (tricobezoar) excised from the duodenum.

On the seventh day after admission, she was transferred to our pediatric surgical ward where she underwent laparotomy with big trichobezoar (8 × 6 cm) removal through an enterotomy (Figure 2) followed by remodeling of dilated jejunum loop. After surgery, blood examinations showed normal serum proteinemia (7.2 g/dl) and stable levels of albumin and hemoglobin (3.5 and 10.6 g/dl, respectively). Moreover, stool alpha-1-antitrypsin levels were markedly reduced (282 mcg/g) confirming the hypothesis that a protein losing enteropathy was the cause of the hypoproteinaemia. During the post-operative recovery, no complications were observed. She was started on total parenteral nutrition for the first few days followed by re-introduction of oral foods which were well-tolerated. The girl underwent psychological evaluation before discharge, while both gastro-nutritional and neuropsychiatric follow-up were planned. She was discharged on day 20 in stable conditions and with normalization of clinical and biochemical nutritional parameters. At 3-month clinical follow-up, the girl has a good nutritional status and no gastrointestinal symptoms; her body weight was 19.8 kg (>3rd percentile), while her height was 112.5 cm (<3rd percentile).

### Discussion

Protein-losing enteropathy (PLE) is a rare condition characterized by protein loss through the gastrointestinal tract, leading to reduced serum protein levels. The most affected protein is albumin, but also other proteins with a slow turn-over rate such as immunoglobulins and ceruloplasmin can be loss. Edema, ascites, pleural, and cardial effusions might complicate hypoproteinemia (3, 4).

The gastrointestinal abnormalities that may cause PLE are the following: (1) abnormalities of the lymphatic system, resulting in leakage of protein-rich lymph as in primary intestinal lymphangiectasia or secondary to obstruction (neoplasm, large bezoars) or elevated lymph pressure (congestive heart failure or after Fontan operation); (2) mucosal injury, resulting in increased mucosal permeability due to mucosal erosion (IBD like Crohn's disease and ulcerative colitis, certain enteric infections with bacteria like Salmonella and Shigella) or non-ulcerative disease (celiac disease, Méntrier é 's disease, allergic gastroenteritis) (3).

The diagnosis of PLE is suspected from clinical manifestations and physical examination and it can be confirmed by the detection of increased level of alpha-1-antitrypsin in a stool sample as observed in our patient (4). The elevated alpha-1-antitrypsin clearance is due to its passage from the blood to the intestinal lumen from where it is not reabsorbed. However, its detection in stool sample does not localize the site of mucosal injury and protein loss (3, 4).

In our case, a protein-losing enteropathy was the presenting manifestation of a large trichobezoar causing bowel obstruction and malabsorption: the girl had a clinical onset characterized by hypo-proteinemia causing a generalized oedema and microcytic hypochromic anemia.

Trichobezoar is a compact and undigested foreign body of swallowed hair usually retained in the gastrointestinal tract, classified as Rapunzel Syndrome (RS) when extending from the stomach, the most frequent location, into the small bowel, which constitutes <6% of all bezoars. This condition is commonly observed in adolescents, particularly in young females with trichotillomania and trichophagia (1, 2), and in patients with gastric motility disturbance. Our patient suffered both from behavioral disorders and possible gut motility impairment, as she had undergone intestinal resection for jejunal atresia and intestinal malrotation during her first year of life. We can speculate that previous surgery might have had an influence on intestinal dysmotility and secondly on trichobezoar formation.

Clinical manifestations of trichobezoar depend on their size: at first patients may be asymptomatic until it reaches a large size; as the trichobezoar enlarges, symptoms develop and may include abdominal pain, nausea, vomiting, early satiety, and anorexia which in association with malabsorption result in malnutrition and macro- an micro nutrient deficiencies. Rarely, gastric bleeding and gastrointestinal obstruction may be observed as complications of large trichobezoar (1, 2, 5, 6). Bezoars are a rare cause of bowel obstruction occurring in <1% of patients with intestinal obstruction (6, 7) and in these circumstances they can cause intestinal plasma protein loss through the following mechanisms: (1) secondary intestinal lymphangiectasia due to mechanical obstruction of intestinal lymphatic drainage and (2) mucosal inflammatory injury (3). PLE has been very rarely reported in the literature as a presenting symptom of RS (8, 9).

Diagnosis of trichobezoar is confirmed by radiological studies or endoscopy. Abdominal ultrasound is often not diagnostic; abdominal X-ray and barium studies may be useful to confirm the clinical diagnosis and to localize obstruction of the gastrointestinal tract secondary to a trichobezoar. However, CT scan is the image study of choice for conclusive diagnosis of suspected trichobezoar which appears as an intra-lumen hypodense and heterogeneous mass (6, 10).

In the acute phase, treatment consists of nutrition support with the means of a high protein diet with fat-soluble vitamin supplement and removal of the foreign body. Endoscopic approach is often ineffective especially for large trichobezoars, and surgical removal by gastrotomy or enterotomy is most commonly required. In our patient trichobezoar was too large to be removed during upper endoscopy requiring surgical intervention.

After surgery and gradual re-feeding, normalization of biochemical and nutritional parameters is observed in most of patients (1, 2, 6).

In conclusion, pediatricians should consider trichobezoar in the differential diagnosis of protein-losing enteropathy in young females, especially if a behavioral disorder, such as pica, is reported. Timely diagnosis and treatment are of great importance to avoid a possible fatal outcome. Psychological and psychiatric evaluation and follow-up are also strongly recommended to treat and to prevent relapses.

## Data Availability Statement

All datasets generated for this study are included in the article/supplementary material.

## Author Contributions

MS and AM take responsibility for the manuscript as a whole. ST critically revised the manuscript. All authors equally contributed to the patient's management and to drafting and revising the manuscript including literature search, figures, and references. All authors read and approved the final manuscript.

### Conflict of Interest

The authors declare that the research was conducted in the absence of any commercial or financial relationships that could be construed as a potential conflict of interest.
